# Inflammatory pseudotumor of the thymus: A case report and review of the literature

**DOI:** 10.3892/ol.2014.1895

**Published:** 2014-02-20

**Authors:** ZHEFENG ZHANG, FENG JIN, LIGUANG SUN, HAO WU, BING CHEN, YOUBIN CUI

**Affiliations:** 1 Department of Thoracic Surgery, The First Hospital of Jilin University, Changchun, Jilin 130021, P.R. China; 2 Academy of Translational Medicine, The First Hospital of Jilin University, Changchun, Jilin 130021, P.R. China; 3 Department of Nephrology, The First Hospital of Jilin University, Changchun, Jilin 130021, P.R. China

**Keywords:** inflammatory pseudotumor, thymus, case report, literature review

## Abstract

Inflammatory pseudotumors are rare benign tumors consisting of cellular and stromal elements of a localized reactive process. While inflammatory pseudotumors are commonly detected in the lung and occasionally in other organs, only one case of inflammatory pseudotumor of the thymus has been reported in the literature to date. This report presents a 54-year-old male patient with inflammatory pseudotumor of the thymus accompanied by pulmonary inflammation. The patient presented with chest pain and moderate fever for 12 days. Enhanced computed tomography of the thorax revealed an anterior mediastinal solid and cystic mass, which constricted the left brachiocephalic vein accompanied by bilateral lung inflammation and marginal pleural effusion. The patient underwent a median sternotomy for the surgical removal of the mass. Histologically, the resected mass was composed of necrotic and fibrous tissues and inflammatory infiltrates, and the diagnosis was confirmed as an inflammatory pseudotumor of the thymus. The patient’s symptoms were resolved following surgery and the patient remained asymptomatic during the six-month follow-up period. In addition, we reviewed the previous literature and discussed the diagnosis and management of our patient. This report provides further insights into the pathogenesis and underlying mechanisms of inflammatory pseudotumors of the thymus to aid in the diagnosis and development of effective therapies.

## Introduction

Inflammatory pseudotumor is a benign, non-neoplastic and tumor-like tissue mass (1). Inflammatory pseudotumor affects both genders and all races, and occurs in patients aged from 1 to 73 years (2,3). Inflammatory pseudotumor mainly arises in the lung and the orbit, but can occur in a variety of organs, including the thyroid, pleura, liver, kidney, common bile ducts, spinal cord, testis, scrotum and other soft tissues (4–7). To the best of our knowledge, only one case of inflammatory pseudotumor of the thymus has been reported in the literature to date (8). Generally, due to the complexity of the mediastinum, inflammatory pseudotumor of the thymus is commonly confused with thymoma and difficult to diagnose, particularly when it causes inflammation in the surrounding organs.

This report presents a case of inflammatory pseudotumor of the thymus, which caused bilateral reactive pulmonary inflammation and pleural effusion. To the best of our knowledge, this is the second case of inflammatory pseudotumor of the thymus with reactive inflammation spreading to the lung. In this study, we describe the diagnosis and treatment of the present case, and discuss the potential factors contributing to the development of pseudotumors. The patient provided written informed consent.

## Case report

A 54-year-old male was referred to The First Hospital of Jilin University (Changchun, China) complaining of chest pain and intermittent degrees of irregular fever, night sweats, morning phlegm (without bleeding) and dysphagia for 12 days. The patient had visited a local clinic and received anti-inflammation treatment one week earlier. Although his fever had been temporarily resolved for two days, symptoms recurred three days ago. The patient had no history of chronic disease, surgery, regular smoking, exposure to occupation-related industry dust or recent travel to other cities. The patient did not show obvious weight loss and none of the patient’s family members had a history of similar symptoms and signs.

Physical examination of the patient revealed the following: Temperature, 38.5°C; heart rate, 75 beats per min; respiration rate, 26 breaths per min; and blood pressure, 135/75 mmHg. Bilateral lymphadenopathy was detected in the neck, but not in the axillary and inguinal lymph nodes. The left enlarged node was ~8×3 mm in size, while the right enlarged node was ~9×3 mm in size. The nodes had an intermediate degree of hardness and tension, but without obvious pain in response to touch. The thorax appeared symmetrical, the intercostal space was bilaterally normal and, on auscultation, no abnormal breath sounds were observed.

Laboratory tests revealed no abnormal changes in full blood counts, differential counts or the concentrations of serum alkaline phosphatase, blood lipids, transaminase, urea nitrogen and creatinine. The patient displayed negative responses to the purified intermediate protein derivative of tuberculin.

**Figure 1:**
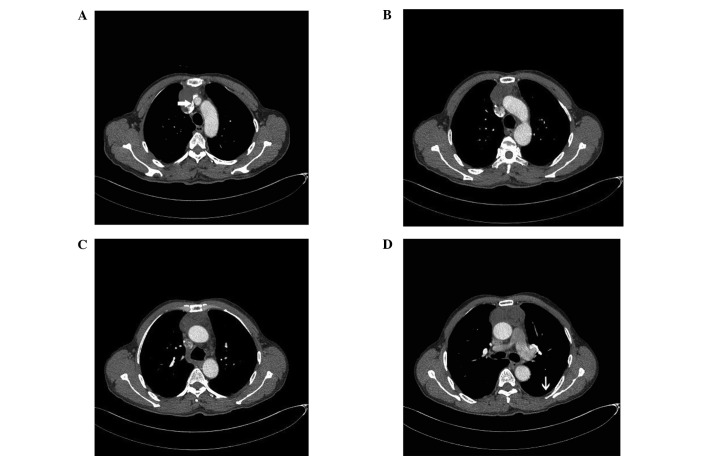
Enhanced computed tomography shows an anterior mediastinal mass (8.3×6×3.5 cm) with heterogeneous contrast enhancement, with an unclear plane separating it from (A) the constricted left innominate vein (white arrow) and (B and C) the aorta and the superior vena cava, and with (D) a trace of pleural effusion (white arrow).

Enhanced computed tomography (CT) revealed an anterior mediastinal irregular solid and cystic mass of ~8.3×6×3.5 cm (Fig. 1), extending posteriorly towards the left innominate vein with heterogeneous enhancement. This was accompanied by an unclear plane separating the mass from the aorta and the superior vena cava, several inflammatory sites in both sides of the lung and a trace of pleural effusion.

**Figure 2:**
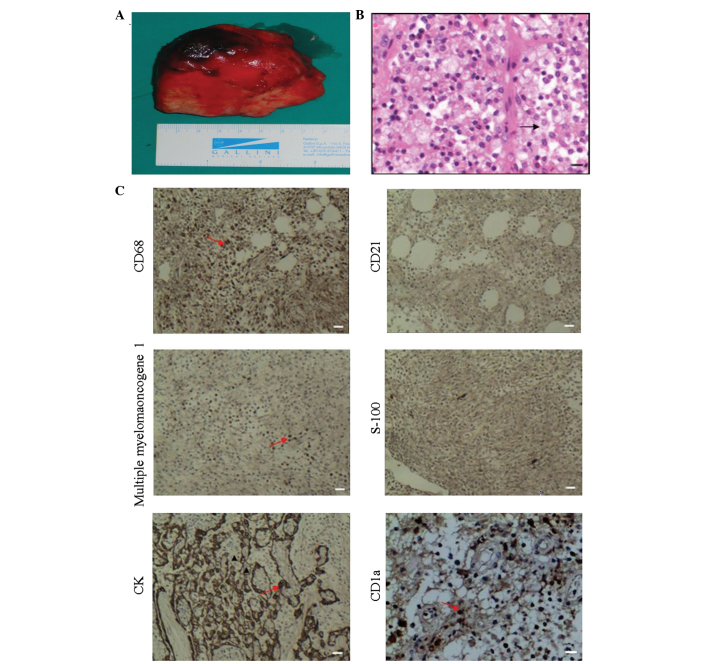
Histological analysis of the mass. The mass (8.3×6.0×3.5 cm) was surgically removed from the mediastinal pleura and subjected to histological and immunohistochemical analyses. Briefly, tissue sections were stained with H&E or anti-CD68, -CD21, -MUM-1, -S-100, -CK or-CD1a overnight at 4°C. The control sections were treated with the same isotype IgG or sera from healthy animals. Subsequently, the bound antibodies were detected with horseradish peroxidase-conjugated secondary antibodies and visualized with 3,3′-diaminobenzidine. (A) Photograph of the gross mass; (B) H&E staining of the tissue section (magnification, ×200: scale bar, 50 μm). (C) Immunohistochemical analysis of CD68, CD21, MUM-1, S-100 and CK (magnification, ×100; scale bar, 20 μm), and CD1a (magnification, ×200; scale bar, 50 μm). Data are representative images and the control sections show no specific staining (data not shown). H&E, hematoxylin and eosin; CD, cluster of differentiation; MUM, multiple myeloma oncogene; CK, cytokeratin.

Accordingly, the patient was suspected to have a thymoma, thymic carcinoma or teratoma. Given the diagnostic uncertainty and the persistent symptoms, the patient was subjected to a median sternotomy. This revealed an ~8.3×6×3.5 cm, hard, well-circumscribed, irregular mass that almost replaced the whole thymus, extending towards the thyroid and posteriorly to the aortico-pulmonary window. The mass invaded the bilateral parietal pleura, pericardium and adhered to the roots of the aorta and superior vena cava. The mass was carefully freed from the left and right mediastinal pleura, and the dorsal aspect of the tumor was separated from the ascending aorta, superior vena cava and left innominate vein, as the tumor was loosely adherent to these. The entire tumor was resected with the bilateral mediastinal pleura and part of the pericardium (Fig. 2A). The mass appeared to be irregular in shape and was covered by a light-yellow, thick fibrotic membrane-like tissue.

During surgery, the mass was considered to be a thymoma or thymic carcinoma. However, histological examination revealed that the tissue sections contained a number of necrotic regions, connective tissue fiber hyperplasia, numerous inflammatory cell infiltrates and an aggregation of foam cells (Fig. 2B). There were no obvious cell characteristics of thymoma or thymic carcinoma. Immunohistochemical analysis revealed that the tissue sections contained a number of cluster of differentiation (CD)68^+^, multiple myeloma oncogene (MUM)-1^+^, cytokeratin (CK)^+^ and CD1a^+^ cells, but there was no detectable anti-S-100 or anti-CD21 staining (Fig. 2C). These findings, together with the inflammatory reactive responses in the lung, aided the diagnosis of inflammatory pseudotumor of the thymus.

The patient was treated postoperatively with 2 g ceftezole sodium twice per day for seven days. Symptoms were resolved and the patient was discharged from hospital without any obvious complications. During the six-month follow-up period, the patient did not experience fever, night-sweats or chest pain and there was no evidence of relapse or lung inflammation.

## Discussion

Inflammatory pseudotumor is a rare disease and is characterized by the excess growth of inflammatory cells. The majority of inflammatory pseudotumors occur in the lung, although there have been cases in other organs, such as the thyroid, pleura, gastrointestinal and central nerve systems, spinal cord, kidney, testis, scrotum and other soft tissues (4–7). Patients with an inflammatory pseudotumor commonly exhibit no specific symptoms, and their clinical symptoms and signs are dependent on the location and size. To the best of our knowledge, this report describes the second case of inflammatory pseudotumor of the thymus. The patient presented with low degrees of irregular fever and night sweats, similar to those experienced in the previous case (8). The patient also complained of morning phlegm without bleeding, as well as dysphagia, but these symptoms were not present in the previous case (8). By contrast, the patient in the present report did not experience myalgias or dyspnoea, as observed in the previous case. Furthermore, the previous case had a significantly elevated serum alkaline phosphatase level; however, this was normal in the present case. This discrepancy may stem from the severity of pulmonary reactive inflammation. Indeed, the previous case had a large amount of yellow exudate in the pleura and diverse inflammation in the lung, whereas our case only showed marginal pleural effusion and mild pulmonary inflammation. Notably, these clinical symptoms may occur in patients with several types of upper respiratory infection and malignancies, such as the early stage of pneumonia, tuberculosis and lung cancer. Therefore, patients with an inflammatory pseudotumor of the thymus can present with a variety of clinical symptoms, which may increase the difficulty in diagnosing inflammatory pseudotumors in the clinic. Physicians assessing patients with these symptoms should consider the possibility of an inflammatory pseudotumor.

Epidemiological investigations have revealed that inflammatory pseudotumors occur in male and female patients at a variety of ages. A previous study showed that almost 25% of cases with inflammatory pseudotumors are individuals <18 years of age (9). The currently available cases are distributed worldwide and there are no significant differences in the geographic distribution. Although we should not exclude the possibility of previous misdiagnosis, to the best of our knowledge, this is the first case of inflammatory pseudotumor of the thymus to be reported in China. Due to the nature of this rare disease, currently there is no information regarding the prevalence and incidence of inflammatory pseudotumor worldwide.

Inflammatory pseudotumors are difficult to diagnose preoperatively and may present with various clinical and radiological characteristics. The diagnosis of an inflammatory pseudotumor is based on histopathological and immunohistochemical examinations. Histological findings include acute and chronic inflammatory infiltrates with varying degrees of fibrosis (5,10). Radiological examination of the present case revealed a mediastinal mass, multiple sites of pulmonary inflammation and pleural effusion. Accordingly, our patient was wrongly diagnosed with thymoma, thymic carcinoma or teratoma preoperatively. Thymoma is a neoplasm in the anterior mediastinum and is frequently associated with indolent growth and a variety of paraneoplastic syndromes, such as myasthenia gravis. Thymomas appear to have malignant potential and should be considered in the differential diagnosis of a mediastinal mass (11,12). Histologically, thymoma usually displays neoplastic epithelial cells with spindle- and/or oval-shaped nuclei. Thymoma cells may also have a dendritic or plump (epithelioid) appearance (13). Although our preoperative diagnosis was understandable, the misdiagnosis may have been prevented by considering the short disease period with no obvious systemic deterioration and the multiple sites of mild pulmonary lesions. Histologically, inflammatory pseudotumors are usually composed of plasma cell granulomas, pulmonary xanthomas, xanthogranulomas, xanthomatous pseudotumor, fibrous histocytomas, plasmacytoma and others (5). The mass sections from our case contained a number of necrotic regions, connective tissue fiber hyperplasia, numerous inflammatory infiltrates and an aggregation of foam cells, which is similar to the mass in the previous case (8). There were no obvious cell characteristics of thymoma or thymic carcinoma. Notably, immunohistochemistry revealed that the tissue sections contained a number of CD68+, MUM-1+, CK+ and CD1a+ cells, but there was no detectable anti-S-100 or anti-CD21 staining. These findings support the diagnosis of an inflammatory pseudotumor of the thymus.

Currently, a complete surgical resection of the inflammatory pseudotumor remains the best treatment. Other non-surgical therapeutic modalities, such as radiotherapy, chemotherapy and steroids, may be useful for individuals with an incomplete surgical resection, multifocal disease, tumor recurrence or contraindication to lung resection (14–16). As the mediastinal mass was completely removed, anti-inflammatory treatment was only provided for a short period. The patient recovered soon after surgery without any surgical complications and no recurrence or other relevant abnormalities were observed during the six-month follow-up period. Our findings suggest that when the inflammatory pseudotumor is completely removed by surgery, it may be not necessary to use other tumor-related therapies.

The number of available studies regarding the etiology of inflammatory pseudotumors is currently limited. Previous studies have suggested that the development of an inflammatory pseudotumor is non-specific in terms of inflammatory reactions, autoimmune responses, trauma or paraneoplastic syndrome (9,17,18). In addition, infection may contribute to the development of an inflammatory pseudotumor and these infectious micropathogens may include influenza, measles, cytomegalovirus and other herpes viruses, mycobacterium tuberculosis, syphilis, brucellosis and Kawasaki disease. Although these infections may result in thymitis, which is associated with the development of an inflammatory pseudotumor of the thymus (8,19), the present case showed no evidence of infection with any of these micropathogens. However, we should not exclude the possibility of unknown endogenous or exogenous viral infection in our patient.

In summary, this report describes a case of inflammatory pseudotumor of the thymus. The patient displayed no specific clinical symptoms or signs and an enhanced CT scan revealed a mediastinal mass and multiple sites of mild pulmonary inflammation. The patient also exhibited marginal pleural effusion, which led to the confusion with thymoma and other solid tumors in the thymus. However, histological and immunohistochemical analysis provided evidence of inflammation, but not neoplastic changes in the thymus sections, and surgical resection of the full mass resulted in a resolution of clinical symptoms and reactive pulmonary responses. Therefore, we propose that physicians should consider an inflammatory pseudotumor when a patient presents with unexplained fever, night sweats and chest pain.
